# Portal Vein Thrombosis: A Rare Finding in a Noncirrhotic Patient

**DOI:** 10.5005/jp-journals-10018-1097

**Published:** 2014-01-22

**Authors:** Aswini Kumar Sahoo, Sudhasmita Rauta, Subash Chandra Mohapatra

**Affiliations:** 1 Department of Medicine, Maharajah’s Institute of Medical Sciences, Nellimarla, Andhra Pradesh, India; 2 Department of Pathology, Maharajah’s Institute of Medical Sciences, Nellimarla, Andhra Pradesh, India; 3 Department of Medicine, Maharajah’s Institute of Medical Sciences, Nellimarla, Andhra Pradesh, India

**Keywords:** Abdominal pain, Hyperhomocysteinemia, Portal vein thrombosis.

## Abstract

Portal vein thrombosis (PVT) is a rare cause of abdominal pain, typically associated with cirrhosis or thrombophilia. A 18-year-old female presented with abdominal pain for 10 weeks. The diagnosis was confirmed with contrast-enhanced computed tomography (CECT) abdomen after an ultrasound showed dilated and obstructed portal vein. This unexpected finding prompted investigation for intrinsic hepatic disease and potential hypercoagulable disorders. Laboratory analysis revealed an elevated serum homocysteine level, an identified risk factor for venous thrombosis. Current literature describes the following factors as indications for anticoagulation: acute thrombus, lack of cavernous transformation, absence of esophageal varices and mesenteric venous thrombosis. PVT is an uncommon cause of abdominal pain, and in the absence of hepatic disease should raise the index of suspicion for an underlying thrombophilia.

**How to cite this article:** Sahoo AK, Rauta S, Mohapatra SC. Portal Vein Thrombosis: A Rare Finding in a Noncirrhotic Patient. Euroasian J Hepato-Gastroenterol 2014;4(1):55-57.

## CASE REPORT

**Fig. 1:**
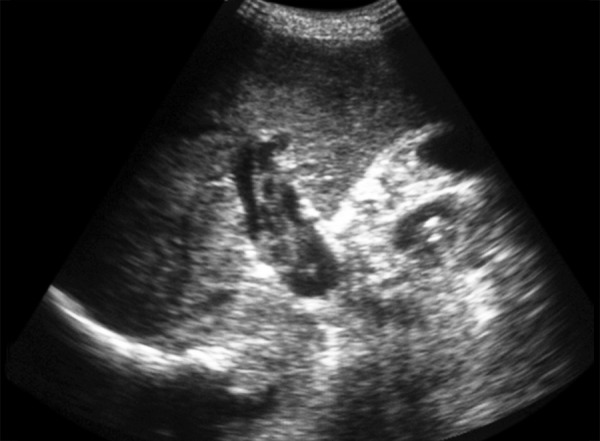
Ultrasonography showed dilated and obstructed portal vein

**Fig. 2:**
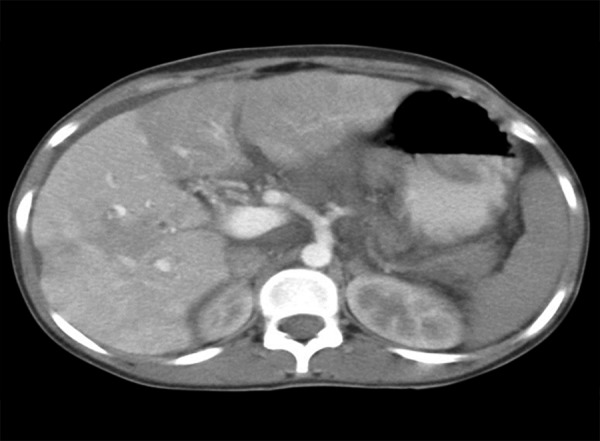
Computed tomography scan shows an enlarged portal vein with multiple linear filling defects extending into the main portal vein

**Fig. 3:**
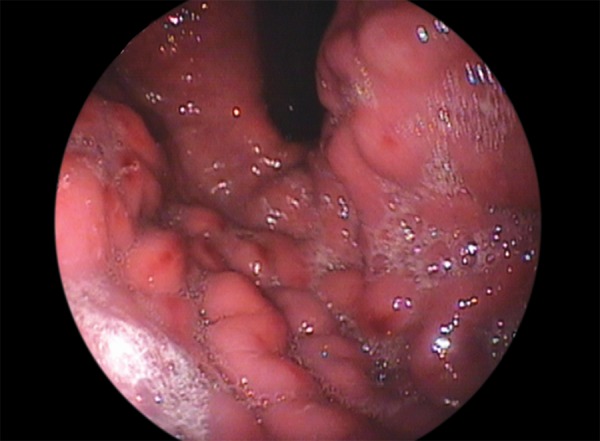
Endoscopy revealed four columns of grade III nonbleeding esophageal varices

**Fig. 4:**
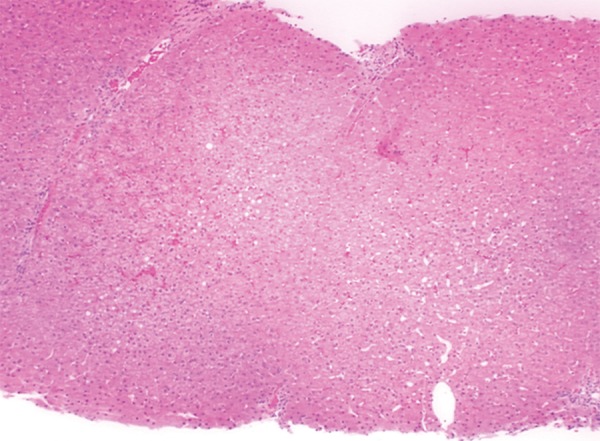
A liver biopsy showed no evidence of cirrhosis

An 18-year-old female presented with 10 weeks history of pain abdomen and 4 kg weight loss. She had no significant medical history. Patient denied consuming alcohol, smoking, consuming tobacco or any drug usage, oral contraceptive pill. Her menstrual history was normal. She was negative for any history of hepatitis, bleeding or clotting disorders. Physical examination reveals mild tender hepatosplenomegaly. She was not icteric. Rectal examination was negative for gross blood. Laboratory findings revealed normal blood counts and transaminase activity. Stool also tested negative for occult blood. An abdominal ultrasound demonstrated mild hepatomegaly with normal echo-texture, mild splenomegaly and the presence of a dilated and obstructed portal vein (Fig. 1). This finding was further confirmed with a computed tomography (CT) scan, which showed an enlarged portal vein (20 mm), with multiple linear filling defects extending into the main portal vein and both the branches with necrosis of segment III of the left lobe of liver (Fig. 2). The scan further described thrombosis into the superior mesenteric vein as well. In light of the finding of PVT, the patient underwent esophagogastroduodenoscopy, which revealed four columns of grade III nonbleeding esophageal varices in the distal esophagus along with fundal gastropathy (esophagogastric varices, type 1) (Fig. 3). Variceal band ligation (VBL) was performed as primary prophylaxis. With an unknown etiology for portal vein thrombosis (PVT), additional laboratory serum studies were performed to search for hypercoagulable disorders as well as evidence of intrinsic hepatic disease. Serum homo-cysteine levels were elevated to 36.94 **m**mol/l and protein S activity was slightly decreased at 54%. A liver biopsy showed no evidence of cirrhosis (Fig. 4). She was also put on propranolol 40 mg/day, vitamin B12, vitamin B6 and folic acid supplementation.

## DISCUSSION

Portal vein thrombosis as the etiology of abdominal pain in an otherwise healthy young adult is an uncommon occurrence. It is most commonly associated with cirrhosis, with PVT ranging from 11 to 6% in known cirrhotic patients.^1,2^ Outside the realm of cirrhosis, PVT is thought to be so rare in the general population that its prevalence has not been defined. Known etiologies can be divided into two broad categories. It has been estimated that in non-cirrhotic PVT patients, thrombophilic states account for approximately 40 to 60% of PVT cases, and local factors are thought to be the causative factor in 10 to 50%.^3,4^ Since, the 1980s and the discovery of several acquired thrombophilic factors, such as hyperhomocysteinemia, several authors have reported a decreasing proportion of idiopathic PVT ranging between 8 and 15%.^3,5^ Specifically regarding PVT, hyperhomocysteinemia has been shown to be the only explainable cause of PVT in a number of cases that would otherwise be named idiopathic. Important consequences of chronic PVT have been primarily related with bowel ischemia and bleeding from esophageal varices, with the latter thought to manifest in 30% of PVT cases.^6^ Retrospectively, Kocher et al^7^ found hyperhomocysteinemia to be present in 12% of patients presenting with a prior diagnosis of idiopathic PVT. Varices with established PVT are often large, which is an independent risk factor for rebleeding risk.^7^ However, it has been objectively documented that large varices secondary to PVT have lower frequency of bleeding, when treated similarly compared with patients, with similar grade varices secondary to liver cirrhosis, 0.25 and 20 to 30% of rebleeding respectively.^7^ These differences are thought to be primarily because of the overall morbidity and complications of an intrinsic liver disease *vs* an isolated and often reversible prothrombotic state. Treatment for varices secondary to PVT is similar to varices from other portal hypertension processes and employs endoscopy eradication with VBL and beta-blockers. One study suggests that these techniques are of low risk and highly successful, demonstrating a 5-year survival rate of 95% and no mortality related to recurrent bleeding in extrahepatic portal vein obstruction.^8^

## CONCLUSION

This case report serves as a reminder that a common presenting symptom, such as abdominal pain in a young, healthy adult can be a manifestation of a rare diagnosis, such as PVT. Literature supports that PVT outside the realm of liver cirrhosis is typically because of a hypercoagulable state, which warrants a methodic search for the specific thrombo-philic etiology. Hyperhomocysteinemia is a relatively new discovery that has been established in the literature as a risk factor for venous thrombosis.

## References

[1] Sheen CL, Lamparelli H, Milne A, Green I, Ramage JK (2000). Clinical features, diagnosis and outcome of acute portal vein thrombosis.. Q J Med.

[2] Webster GJM, Burroughs AK, Riordan SM (2005). Review article: portal vein thrombosis—new insights into aetiology and management.. Aliment Pharmacol Ther.

[3] Valla DC (1999). Portal vein thrombosis and prothrombic disorders.. J Gastroenterol Hepatol.

[4] Hegenbarth K, Fickert P, Aschauer M, Horina JH, Stauber RE, Trauner M (2002). Successful management of acute portal vein thrombosis by low molecular weight heparin and oral anticoagulation.. Am J Gastroenterol.

[5] Okuda K, Ohnishi K, Kimura K (1985). Incidence of portal vein thrombosis in liver cirrhosis. An angiographic study in 708 patients.. Gastroenterology.

[6] Denninger MH, Chait Y, Casadevall N (2000). Cause of portal or hepatic venous thrombosis in adults: the role of multiple concurrent factors.. Hepatology.

[7] Kocher G, Himmelmann A (2005). Portal vein thrombosis (PVT): a study of 20 noncirrhotic cases.. Swiss Med Wkly.

[8] Choudhary A, Grayer D, Nelson A, Roberts I (2000). Mesenteric venous thrombosis—a diagnosis not to be missed.. J Clin Gastroenterol.

